# A Social Media–Based Diabetes Intervention for Low-Income Mandarin-Speaking Chinese Immigrants in the United States: Feasibility Study

**DOI:** 10.2196/37737

**Published:** 2022-05-11

**Authors:** Lu Hu, Nadia Islam, Chau Trinh-Shevrin, Bei Wu, Naumi Feldman, Kosuke Tamura, Nan Jiang, Sahnah Lim, Chan Wang, Omonigho M Bubu, Antoinette Schoenthaler, Gbenga Ogedegbe, Mary Ann Sevick

**Affiliations:** 1 Center for Healthful Behavior Change Institute for Excellence in Health Equity NYU Langone Health New York, NY United States; 2 Department of Population Health NYU Grossman School of Medicine New York, NY United States; 3 Rory Meyers College of Nursing New York University New York, NY United States; 4 Charles B Wang Community Health Center New York, NY United States; 5 Socio-Spatial Determinants of Health (SSDH) Laboratory, Population and Community Health Sciences Branch Intramural Research Program, National Institute on Minority Health and Health Disparities National Institute of Health Bethesda, MD United States; 6 Department of Psychiatry NYU Grossman School of Medicine New York, NY United States; 7 Department of Medicine NYU Grossman School of Medicine New York, NY United States

**Keywords:** diabetes, health equity, immigrant health, migrant, minority, mobile health, social media, WeChat, messaging app, patient education, health education, education video, health video, counseling, pilot study, feasibility, access to care, diabetes education, disease management, self management, low income, immigrant population, digital health, health intervention, mobile phone

## Abstract

**Background:**

Chinese immigrants bear a high diabetes burden and face significant barriers to accessing diabetes self-management education (DSME) and counseling programs.

**Objective:**

The goal of this study was to examine the feasibility and acceptability and to pilot test the potential efficacy of a social media–based DSME intervention among low-income Chinese immigrants with type 2 diabetes (T2D) in New York City.

**Methods:**

This was a single group pretest and posttest study in 30 Chinese immigrants with T2D. The intervention included 24 culturally and linguistically tailored DSME videos, focusing on diabetes education and behavioral counseling techniques. Over 12 weeks, participants received 2 brief videos each week via WeChat, a free social media app popular among Chinese immigrants. Primary outcomes included the feasibility and acceptability of the intervention. Feasibility was evaluated by recruitment processes, retention rates, and the video watch rate. Acceptability was assessed via a satisfaction survey at 3 months. Secondary outcomes, that is, hemoglobin A_1c_ (HbA_1c_), self-efficacy, dietary intake, and physical activity, were measured at baseline, 3 months, and 6 months. Descriptive statistics and paired 2-sided *t* tests were used to summarize the baseline characteristics and changes before and after the intervention.

**Results:**

The sample population (N=30) consisted of mostly females (21/30, 70%) who were married (19/30, 63%), with limited English proficiency (30/30, 100%), and the mean age was 61 (SD 7) years. Most reported an annual household income of <US $25,000 (24/30, 80%) and a high school education or less (19/30, 63%). Thirty participants were recruited within 2 months (January and February 2020), and 97% (29/30) of the participants were retained at 6 months. A video watch rate of 92% (28/30) was achieved. The mean baseline HbA_1c_ level was 7.3% (SD 1.3%), and this level declined by 0.5% (95% CI –0.8% to –0.2%; *P*=.003) at 6 months. The mean satisfaction score was 9.9 (SD 0.6) out of 10, indicating a high level of satisfaction with the program. All strongly agreed or agreed that they preferred this video-based DSME over face-to-face visits. Compared to baseline, there were significant improvements in self-efficacy, dietary, and physical activity behaviors at 6 months.

**Conclusions:**

This pilot study demonstrated that a social media–based DSME intervention is feasible, acceptable, and potentially efficacious in a low-income Chinese immigrant population with T2D. Future studies need to examine the efficacy in an adequately powered clinical trial.

## Introduction

In the United States, the Chinese immigrant population is a fast-growing minority group and bears a disproportionately high type 2 diabetes (T2D) burden compared to the general adult population [1,2]. In New York City, data from an epidemiological survey of 2071 participants revealed that almost 1 out of every 2 adult Chinese immigrants has T2D or impaired fasting glucose [2]. This is a concerning rate, given the high proportion and the continuing rapid influx of Chinese immigrants over the last few decades. Indeed, between 2000 and 2015, the New York City Chinese population grew by 49% (increased from 260,928 in 2000 to 388,783 in 2015) compared to 12% in the overall New York City immigrant population (increased from 2.87 million in 2000 to 3.21 million in 2015) [3]. Although the poverty rate of Chinese Americans in New York City is comparable to that of the overall New York City population, the older Chinese adults (28.6%) who are more likely to be impacted by diabetes have a much higher poverty rate relative to the overall older adults (18.8%) in New York City [4].

Despite the high T2D burden, research in this patient population is quite limited, with only few diabetes intervention studies identified [5-8]. In each study, the interventions relied on busy health care providers or dedicated study staff to deliver them, thereby limiting their sustainability and scalability. Each intervention required frequent travel to a central location to receive the intervention (6-12 face-to-face sessions lasting 1-2 hours), which is unlikely to be feasible in a real-world setting, especially among low-income Chinese immigrants, given their long work hours [9,10].

Diabetes self-management education (DSME) and counseling programs are evidence-based interventions that provide important knowledge, skills, and counseling to patients with T2D [11]. Chinese immigrants face numerous barriers to accessing these in-person programs [8,10,12-15], and these programs have proven to be effective in mostly English-speaking populations [16-18]. The lack of linguistically and culturally sensitive health care providers and tailored diabetes programs have been cited as the major reasons for poor diabetes outcomes in Chinese Americans [13,19-21]. While over 60% of Chinese immigrants report limited English proficiency in New York City [3], language-concordant providers are also limited in number [13,19-21]. Differing cultural norms also complicate the delivery of effective diabetes care and counseling [10,13-15,22]. These cultural and linguistic discordances between providers and patients often contribute to poor patient-provider communication, inefficient diabetes education and counseling, and diminished understanding and confidence in managing T2D at home [21]. Moreover, these DSME programs are often labor-intensive for providers and time-consuming for patients [9,23]. Chinese immigrants are often engaged in low-wage jobs with long working hours, limited sick leave, and lack of health care insurance [4], which prevent them from participating in these in-person multiple-session programs [9,10,22].

Leveraging widely used social media platforms may be a promising approach to deliver DSME to underserved Chinese immigrants in a time and place that is convenient to them [24,25]. In a prior study, we demonstrated ownership of smart devices by a majority of Chinese immigrants with T2D, widespread use of the free social media app, “WeChat,” and a strong interest in a WeChat-based DSME [26]. Indeed, over 90% of Chinese immigrants with T2D reported owning a smart device and more than 70% currently use WeChat [26]. Given the ubiquitous nature of mobile phones, a social media–based asynchronous intervention holds strong promise to be integrated into the daily lives of Chinese immigrants. The goal of this pilot study was to examine the feasibility, acceptability, and potential efficacy of an asynchronous WeChat-based DSME program in Chinese immigrants with T2D.

## Methods

### Study Design

The CARE (Chinese American Research and Education) study was a pretest and posttest single-group study on 30 Chinese immigrants with T2D. During the 12 weeks of the intervention, each participant received links to 2 diabetes videos via WeChat each week. Participants completed in-person surveys at enrollment (prior to the intervention) and 3- and 6-month follow-up surveys over the telephone. All participants provided written informed consent.

### Ethics Approval

This study was approved by the New York University Grossman School of Medicine Institutional Review Board (s18-00609).

### Participants

Participants were eligible if they (1) self-identified as a Chinese immigrant, (2) were between 18 and 70 years old, (3) self-reported a diagnosis of T2D, (4) were able to understand Mandarin Chinese, (5) were currently using WeChat, (6) had a smartphone/tablet or were willing to use a study smartphone, and (7) reported interest in receiving diabetes videos via WeChat. Exclusion criteria included pregnancy, breastfeeding, or living in nursing homes or facilities in which participants had limited opportunity to engagement in independent decision-making regarding management of their T2D. We chose to focus on Mandarin in this study because this is among the most popular spoken languages among New York City Chinese immigrants [27].

### Recruitment, Screening, and Baseline Assessment

Participants were recruited from a diabetes research registry that has been established from one of our prior studies [26], which aimed to examine diabetes self-management behaviors in Chinese immigrants with T2D in New York City. Study staff contacted potential participants, explained the study, and screened them for eligibility. Once eligibility was confirmed, study staff scheduled a date and time to meet in person for written consent and baseline assessment.

### Intervention Overview and Theoretical Frameworks

We culturally and linguistically adapted a DSME intervention shown to be efficacious for decreasing hemoglobin A_1c_ (HbA_1c_) levels in a highly educated non-Hispanic White population in the ENHANCE trial [28]. Adaptation of the ENHANCE intervention was guided by the Cultural Adaptation Model [29] and the Ecological Validity Model [30]. These models were chosen because they have been widely used in the literature to guide the cultural adaptation processes of evidence-based interventions. The study team first reviewed the ENHANCE intervention content to make sure it is consistent with the latest diabetes and DSME guidelines [11] and covers all of the Association of Diabetes Care & Education Specialists self-care behavior topics [31]. Then, informed by the adaptation models and our prior formative work, we tailored the content based on Chinese culture and norms. For instance, when talking about diet, we used commonly consumed Chinese dishes, food items, seasoning, and cooking methods. We also discussed tips to maintain healthy eating during Chinese holidays (eg, Lunar New Year). When discussing medication taking, we shared commonly reported barriers to medication taking in Chinese immigrants and provided tailored strategies to improve medication adherence. Once the initial adaptations were completed, the intervention content was shared with a Chinese diabetes educator and a nurse for feedback on cultural relevance and content accuracy.

CARE included a 12-week intervention program involving DSME videos that participants could access in a time and place convenient to them. We developed 24 brief (~5 minutes) diabetes videos in Mandarin Chinese. Each week, 2 video links were sent to the participants via WeChat, with one video focusing on diabetes education (eg, basics of diabetes care, diet, physical activity) and the second one focusing on social cognitive theory–based behavioral change techniques (eg, goal setting, self-reward, problem solving) (see Table 1 for the outline of the intervention videos) [30,31]. At the end of the baseline visit, participants were sent the first video and shown how to open the video by clicking the play button. Since all participants knew how to use WeChat, we did not provide any guidance around using WeChat. In addition to sending brief videos, study staff called the study participants every 2 weeks to ask if they had questions regarding the video content and gave them an opportunity to discuss any challenges encountered in their diabetes self-management efforts. The phone calls typically lasted about 15 minutes.

**Table 1:** Outline of the intervention content.

Week	Education materials (A)	Social cognitive theory–based behavioral materials (B)
1	Overview of diabetes	Goals for life
2	Healthy diet part 1	Setting goals
3	Healthy diet part 2	Self-reward, turning goals into habits
4	Medication management	Social support, developing and making your social support network
5	Glucose self-monitoring	Problem solving: barriers and setbacks Problem solving model
6	Exercise and diabetes	Problem solving: behavioral triggers and stimulus control
7	Building muscles with strength training	Problem solving: emotional eating
8	Grocery shopping at a Chinese grocery store	Problem solving: cravings for white rice, noodle, bun, dumplings etc
9	Stress and diabetes	Problem solving: eliminating negative self-talk
10	Chinese holidays and eating out	Problem solving: anticipating high-risk situations
11	Attending doctor appointments	Problem solving: lapse and relapse
12	Navigating the US health care system	Problem solving: coping with lapses and setting new goals

### Measures

Unless specified otherwise below, measurements were obtained at baseline, 3 months, and 6 months.

#### Sociodemographic Data

At baseline only, we collected data on age, gender, income, education, marital status, employment status, and English proficiency. English proficiency was measured by 1 question “how well do you speak English,” and limited English proficiency was defined as speaking English less than very well.

#### Primary Outcomes

Feasibility was measured as the percentage of those screened and eligible who enrolled in the study, retention rates at 3- and 6-month follow-up, and the video watch rate. At the end of each video, participants were asked 2 brief questions via WeChat: (1) how much of the video did you watch? (part of the video vs the entire video) and (2) how helpful was the video? (not at all helpful, somewhat helpful, or extremely helpful). If participants responded to these 2 questions, we considered that they watched the video. The number of responses was used as a proxy measure to estimate the video watch rate. Acceptability was measured by a 10-item patient satisfaction scale used in a prior study [32]. Participants were provided with 9 statements regarding their experience and satisfaction to which they provided their level of agreement, using a 5-point Likert scale (1=strongly agree to 5=strongly disagree). Participants also responded to a single 11-point Likert-scaled item reflecting their overall satisfaction with the intervention (0=not at all satisfied to 10=totally satisfied).

#### Secondary Outcomes

##### HbA_1c_ Levels

Baseline HbA_1c_ was abstracted from the medical record if a result was available within 3 months prior to enrollment. If a baseline HbA_1c_ result was not available, point-of-care A1CNOW testing was performed. Follow-up HbA_1c_ was obtained from the electronic record. Because of COVID-19 interruptions in the delivery of ambulatory care, many participants did not seek routine care during the study midpoint (3 months follow-up) and HbA_1c_ data were not available for them. However, ambulatory care delivery returned to normal as the study concluded, and HbA_1c_ data were available for most participants at the 6-month follow-up.

##### Self-efficacy

Self-efficacy was measured with the Stanford 8-item Self-Efficacy for Diabetes scale [33,34] on which participants report confidence in their ability to manage various diabetes self-care behaviors. Each item was rated using a 10-point Likert scale (from 1=not at all confident to 10=totally confident). The final score was the mean across the 8 items, with higher scores reflecting higher self-efficacy.

##### Dietary Intake

Dietary intake was measured with the 8-item Starting The Conversation scale [35], which asks participants to report the frequency with which they consumed various foods and drinks over the past few months (eg, fruits, vegetables, sodas, desserts). The final score was the sum of 8 items, with a possible range of 0-16. Lower scores reflect more healthy eating behaviors.

##### Physical Activity

Physical activity was measured with the International Physical Activity Questionnaire (short version) [36]. Participants were asked whether they engaged in any vigorous, moderate, or mild level of physical activity over the past 7 days and the duration for each exercise intensity. Results were calculated in total MET-minutes/week [37], with higher scores reflecting a higher level of physical activity.

### Statistical Analyses

For primary outcomes, we used descriptive statistics to summarize demographics, retention rates at 3 and 6 months, the video watch rate, and intervention satisfaction scores. For secondary outcomes, we used paired 2-sided *t* tests to identify changes over time. Changes in means and their 95% CIs were presented. We performed all data analyses using SPSS (version 25.0, IBM Corp).

## Results

### Characteristics of the Sample Population

As shown in Table 2, the sample consisted of 30 middle-aged adults or older Chinese immigrants who were primarily married women with a high school education or less, an annual household income of less than US $25,000, and who reported limited English proficiency.

**Table 2:** Sample characteristics (N=30).

Characteristic			Value
Age (years), mean (SD)			61 (7)
**Gender, n (%)**			
	Female	21 (70)	
	Male	9 (30)	
**Marital status, n (%)**			
	Currently married or living as married	19 (63)	
	Divorced or separated	7 (24)	
	Widowed	3 (10)	
	Single or never married	1 (3)	
**Educational attainment, n (%)**			
	High school education or less	19 (63)	
	Some college or technical school	9 (30)	
	College graduate or more	2 (7)	
**Annual household income, n (%)**			
	<US $25,000	24 (80)	
	US $25,000-US $55,000	3 (10)	
	≥US $55,000	2 (7)	
	Declined to answer or don’t know	1 (3)	
**Employment status, n (%)**			
	Employed full time	5 (17)	
	Part-time (one job)	14 (47)	
	Part-time (multiple jobs)	2 (7)	
	Self-employed	1 (3)	
	Not employed, not working	7 (23)	
	Retired	1 (3)	
**English proficiency, n (%)**			
	Very well	0 (0)	
	Well	4 (13)	
	Not well	18 (60)	
	Not at all	8 (27)	
Number of years living in the United States, mean (SD)			13 (7)
Duration of self-report of having type 2 diabetes, mean (SD)			9 (7)

### Feasibility and Acceptability Outcomes

We report feasibility in the following 3 ways.

#### Recruitment

A total of 30 participants were recruited from January 16 to February 27, 2020, prior to the onset of the COVID-19 pandemic in New York City. We called 70 potential patients and were able to reach 45 participants (64%). Of these 45 patients, 38 were eligible and 30 enrolled (79%) in this study. Thus, we needed to screen 1.5 patients to enroll 1 participant in the study (45 screened/30 enrolled).

#### Retention Rates

The retention rate was 100% (30/30) at 3-month follow-up and 97% (29/30) at 6-month follow-up. One participant moved back to mainland China and was lost to follow-up at the 6-month follow-up.

#### Video Watch Rate

The mean video watch rate was 92% (SD 4%), indicating that on average, each video was watched by 92% (28/30) of the sample. The video watch rate over the 12-week intervention ranged from 83% to 100%. Almost all (99.6%) reported watching the entire video when they watched a video and 86% (26/30) agreed that the videos were very helpful.

#### Acceptability

Out of a possible score of 10 with higher scores reflecting greater satisfaction, the mean overall satisfaction with the intervention was 9.9 (SD 0.6). Table 3 summarizes participants’ responses to each satisfaction item. All participants agreed or strongly agreed that it was very easy to receive and view diabetes videos and that the videos provided very helpful information on diet and physical activity. All participants strongly agreed or agreed that the diabetes videos enhanced their confidence to manage their T2D. Of note, all strongly agreed or agreed that they preferred video-based diabetes education over in-person face-to-face education in their doctor’s office.

**Table 3:** Satisfaction survey results (N=30).

To what extent do you agree with the following statements?	Strongly agree, n (%)	Agree, n (%)	Neutral, n (%)	Disagree or strongly disagree, n (%)	Not applicable, n (%)
It was easy to receive and view the WeChat diabetes videos from the research team.	22 (73)	8 (27)	0 (0)	0 (0)	0 (0)
I found this program to be helpful for providing me more information about healthy diet.	27 (90)	3 (10)	0 (0)	0 (0)	0 (0)
I found this program to be helpful for providing me more information about physical activity.	28 (93)	2 (7)	0 (0)	0 (0)	0 (0)
I found this program to be helpful at motivating me to take my diabetes medication as prescribed.	24 (80)	5 (17)	0 (0)	0 (0)	1 (3)
I found this program to be helpful at motivating me to check my blood sugar as recommended.	21 (70)	6 (20)	1 (3)	0 (0)	2 (7)
I found this program to be helpful at increasing my confidence to manage my diabetes.	27 (90)	3 (10)	0 (0)	0 (0)	0 (0)
I would be willing to join similar programs in the future to help me manage my diabetes.	27 (90)	3 (10)	0 (0)	0 (0)	0 (0)
I would recommend this program to my friends/family that have diabetes.	23 (77)	7 (23)	0 (0)	0 (0)	0 (0)
I prefer to receive diabetes education via WeChat than scheduling appointment and going to doctor’s office.	24 (80)	6 (20)	0 (0)	0 (0)	0 (0)

### Secondary Outcomes

Table 4 shows the changes in the secondary outcomes over time. Compared to baseline, there were significant improvements at 3 months in self-efficacy, while no changes were observed in dietary and physical activity behaviors. Between baseline and 6 months, significant improvements were observed in HbA_1c_, self-efficacy, dietary behavior, and physical activity.

**Table 4:** Changes in secondary outcomes over time.

	0 month (baseline), mean (SD)	3 months, mean (SD)	6 months, mean (SD)	0-3 months change (95% CI)	*P* value	3-6 months change (95% CI)	*P* value	0-6 months change (95% CI)	*P* value
Hemoglobin A_1c_	7.3 (1.3)	N/A^a^	6.9 (1.3)	N/A	N/A	N/A	N/A	–0.5 (–0.8 to –0.2)	.003
Self-efficacy (score range: 0-10)	8.0 (1.4)	8.7 (0.8)	8.9 (0.9)	0.7 (0.2 to 1.2)	.01	0.2 (–0.01 to 0.5)	.06	0.9 (0.4 to 1.3)	.001
Dietary intake (score range: 0-16)	5.1 (2.3)	5.2 (2.0)	3.4 (1.7)	0.1 (–0.6 to 0.8)	.79	–1.9 (–2.6 to –1.1)	<.001	–1.7 (–2.5 to –1.0)	<.001
Physical activity (MET-mins/week)	1431.6 (803.6)	1834.0 (1372.8)	2355.3 (1798.1)	404.7 (–290.0 to 1099.5)	.24	623.7 (47.7 to 1199.8)	.04	1008.1 (225.5 to 1790.7)	.01

^a^N/A: not applicable.

## Discussion

### Principal Findings

To the best of our knowledge, this is the first study examining the feasibility and acceptability of leveraging a free social media platform to deliver culturally and linguistically tailored, asynchronous DSME to underserved Chinese immigrants with T2D. The results of this study demonstrated high feasibility and acceptability of this intervention. The retention rate was comparable to or better than that previously reported in in-person diabetes interventions in Chinese immigrants [8,9,38]. The video watch rate was high over the 12-week intervention, with an adherence rate higher than most in-person DSME interventions in Chinese immigrants [6,8,9,38]. For example, in a study of 145 Chinese immigrants with T2D, Chesla and colleagues [8] examined the effect of a 6-session intervention program and found that the cumulative percentages of participants who attended 4, 5, or 6 sessions were 92%, 79%, and 58%, respectively. Similarly, in another in-person diabetes prevention program in Chinese immigrants, the average session attendance was 77% [38]. Several factors may explain the higher engagement observed in this study, including culturally and linguistically tailored intervention content, bilingual study staff, and remote delivery of the intervention via a commonly used social media app to Chinese immigrants, which allows participants to access the intervention videos at a time and place convenient to them. In addition, the study staff called participants every 2 weeks to check whether they had any questions with regard to the video content, which may help build a trusting relationship with participants and thus enhance retention and engagement.

The intervention in this study shows promise for improving glycemic control and key diabetes psychosocial and behavioral outcomes. The reduction in HbA_1c_ levels was both statistically and clinically significant, with an effect size similar to in-person but more labor-intensive DSME programs [39]. We also found that participants reported higher self-efficacy for managing T2D and better adherence to diet and physical activity, which have been considered as critical factors in improving glycemic control and diabetes outcomes [11,40]. It is possible that the effect of our intervention was mediated by improvements in self-efficacy and adherence to self-management behaviors. The small sample size of this study precluded the possibility of mediation analyses. Future large randomized controlled trials may consider exploring the mechanism of this social media–based DSME intervention.

The literature regarding mobile health–based interventions has been rapidly growing over the past few decades. However, most published studies have focused on developing and designing sophisticated applications or technologies to serve well-educated English-speaking populations [41]. Use of mobile technologies to deliver health education to underserved populations, particularly marginalized low-income immigrants with limited English proficiency, is largely untested [41-43]. Our data suggest that leveraging a communication app widely used by an underserved immigrant community is feasible for delivering asynchronous DSME among Chinese immigrants. The intervention is acceptable and promising to improve diabetes outcomes and related health behaviors among low-income Chinese immigrants.

The data in this study need to be interpreted cautiously, and additional research will be required to confirm the results, given the small sample and pretest-posttest study design. In addition, the video watch rate and program satisfaction were measured by self-reported questions that could reflect response bias. Nonetheless, this study is the first to examine the use of a free social media platform to increase access to culturally tailored DSME among underserved Chinese immigrants. Future large-scale randomized controlled trials are needed to explore whether this approach can be used for other chronic disease management interventions or in other high-risk immigrant populations in the United States. Use of a free widely used communication platform enhances future scalability and minimizes potential digital literacy concerns.

### Conclusion

Underserved racial and ethnic minority and immigrant populations bear disproportionately high burden of T2D and face numerous barriers to accessing culturally appropriate DSME. In this study, we demonstrate the feasibility, acceptability, and potential efficacy of an asynchronous intervention that employs a free social media platform to deliver culturally and linguistically tailored DSME videos to underserved low-income Chinese immigrants. These findings add to the scarce literature on the use of mobile health interventions in underserved populations. Future studies are required to confirm the efficacy of the intervention in a randomized controlled trial.

## Abbreviations

CARE: Chinese American Research and Education
DSME: diabetes self-management education
HbA_1c_: hemoglobin A_1c_
T2D: type 2 diabetes
